# A Novel Cost-Effective Technique for Speedy Resolution of Infantile Umbilical Hernia: Ammannaya's Technique

**DOI:** 10.1155/2019/3806358

**Published:** 2019-09-10

**Authors:** Ganesh Kumar K. Ammannaya, Ninada Sripad

**Affiliations:** ^1^Department of Cardiovascular & Thoracic Surgery, Lokmanya Tilak Municipal Medical College & General Hospital, Sion, Mumbai, India; ^2^Department of Microbiology, Goa Medical College, Goa, India

## Abstract

Umbilical hernia in the infant is common and resolves in majority of the cases by 6 years of age. Observation till this age and surgery in the event of persistence are the widely followed management strategies. Trusses, taping, and adhesive strapping have been tried to achieve speedy resolution with variable success and a significant incidence of skin complications. We present a novel, simple, easily reproducible, and highly cost-effective technique to achieve complete resolution of infantile umbilical hernia in a span of 8 weeks, with no skin complications.

## 1. Introduction

Infantile umbilical hernia is a relatively common finding with an incidence of over 10% in most races, being more prevalent in preterm and low-birth weight babies [1]. It is usually identified as an umbilical bulge during the first few months of life, following the separation of the umbilical cord. It consists of a peritoneum-lined sac protruding through the umbilical ring, which is an opening in the deep fascia of the abdominal wall. The cord structures fail to fuse with the umbilical foramen, leaving a patent umbilical ring. The failure of this ring to close results in umbilical hernia [2].

Though largely regarded as a benign condition, infantile umbilical hernia is often a cause of great concern and anxiety to parents and caregivers [3]. Most umbilical hernias spontaneously close by 3 to 5 years of age, and more than 85% close by the age of 6 years [2, 4]. Unless the defect is large (>1.5 cm), spontaneous closure can be anticipated. Therefore, reassurance and observation are the usual management approaches for vast majority of patients. However, any defect that persists beyond the age of 6 years requires surgical repair. Incarceration, although rare, warrants urgent surgical evaluation and repair. Taping the hernia is not recommended and has not been proven to increase the rate of hernia resolution, but, on the contrary, may cause significant skin breakdown [2].

In this article, we present the use of a novel and an extremely cost-effective conservative technique which aided the speedy resolution of an infantile umbilical hernia, with a completely normal looking umbilicus at 8 weeks of treatment.

## 2. Case Report

**Figure 1:**
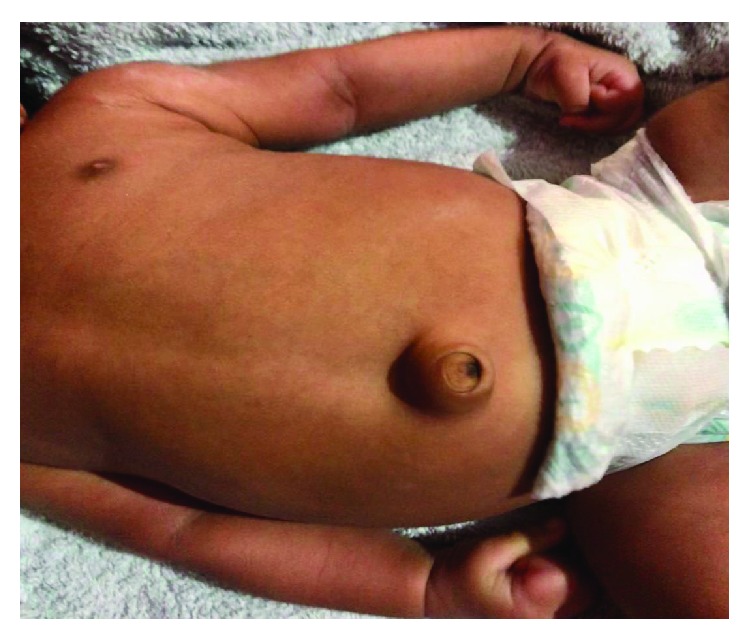
Infantile umbilical hernia.

An infant born at a gestational age of 37 weeks and 4 days through an elective cesarean section and with a birth weight of 2.60 kg presented with umbilical hernia at 6 weeks of age. The child was otherwise healthy with a normal hematological and thyroid profile. On clinical examination, the umbilical hernia was 2.5 × 2.5 cm in dimension (Figure 1), easily reducible with contents as loops of the small bowel. The size of the defect was 1 × 1 cm.

**Figure 2:**
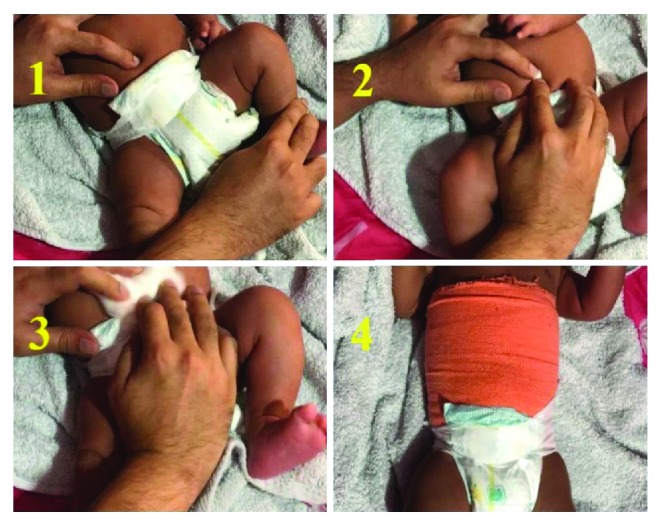
Stepwise technique in the management of infantile umbilical hernia.

**Figure 3:**
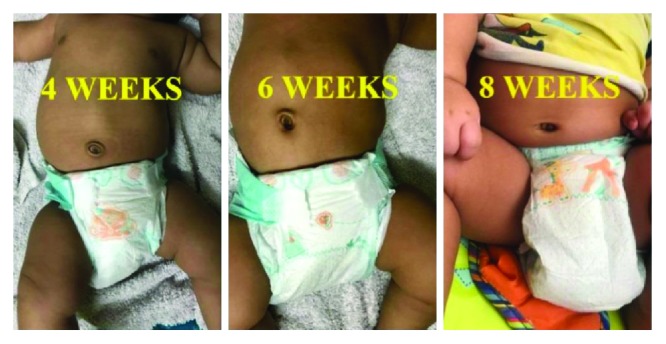
Resolution of infantile umbilical hernia.

The child was treated conservatively through a new technique using an elastic crepe bandage, as summarized in Table 1 and demonstrated in Figure 2. The technique was followed every day maintaining good hygiene, and the crepe bandage was kept on at all times, except daily changing while bathing. A broader (6-inch) crepe bandage was used to prevent displacement and keep the hernia reduced at all times when in use. The resolution of hernia was evident from 4 weeks which started off as umbilical inversion and completed through weeks 6-8, with total disappearance of the hernia at 8 weeks of treatment (Figure 3). Subsequently, the hernia did not reappear on maneuvers increasing intra-abdominal pressure such as crying. Clinical examination confirmed the resolution of hernia, as the defect was no longer palpable.

## 3. Discussion

Infantile umbilical hernia can be a source of great worry for the parents and caregivers until its resolution which may take as long as 6 years. Trusses and abdominal binders have been used with inconsistent results [5, 6]. These have the drawbacks of not being readily available for the infant size and being expensive, costing anywhere between 50 and 150 USD. Taping is strongly discouraged owing to a high incidence of skin breakdown [2]. Other conservative means such as adhesive strapping to hasten resolution as compared to observation alone have been tried previously. These have met with reasonable success, but at the cost of significant skin complications occurring in up to 26% of the treated cases [4].

Here, we present a novel, simple, and reproducible technique to effect a speedy resolution of the umbilical hernia in an infant—“Ammannaya's technique” of conservative management of infantile umbilical hernia. This technique has not been described in literature to date and was successful in achieving complete resolution of a 2.5 × 2.5 cm infantile umbilical hernia with a fascial defect of 1 × 1 cm with 8 weeks of regular treatment. The technique was highly cost-effective, requiring merely 2 USD for its successful application and hence can be particularly appealing in the developing world. The absence of skin breakdown or other skin complications makes this technique greatly preferable to adhesive strapping.

## 4. Conclusion

A simple, highly cost-effective, and easily reproducible technique can achieve a complete resolution of infantile umbilical hernia within a short span of up to 8 weeks and thus save the parents and caregivers from anxiety as well as the need for any future surgery. A clinical study would be greatly helpful to further validate the results of this novel technique.

## Figures and Tables

**Table 1:** The technique of conservative management of infantile umbilical hernia.

Step-by-step technique in the management of infantile umbilical hernia
Step 1: digital reduction of the umbilical hernia
Step 2: placement of a small cotton ball to keep the hernia in the reduced position
Step 3: placement of additional cotton padding for support
Step 4: placement of an elastic crepe bandage. The crepe bandage is rolled around the trunk, covering the reduced umbilical hernia, such that it is neither too tight (should allow at least two fingers to pass beneath) nor too loose, thus maintaining the hernia in a reduced position
